# Did Universal Access to ARVT in Mexico Impact Suboptimal Antiretroviral Prescriptions?

**DOI:** 10.1155/2013/170417

**Published:** 2013-12-11

**Authors:** Yanink Caro-Vega, Patricia Volkow, Juan Sierra-Madero, M. Arantxa Colchero, Brenda Crabtree-Ramírez, Sergio Bautista-Arredondo

**Affiliations:** ^1^National Institute of Public Health, Health Economics Division, Avendia Universidad No. 655, Colonia Santa María Ahuacatitlán, Cerrada Los Pinos y Caminera, 62100 Cuernavaca, MOR, Mexico; ^2^National Institute of Medical Sciences and Nutrition, Salvador de Zubirán Unidad del Paciente Ambulatorio (UPA), 5to Piso Vasco de Quiroga No. 15, Col. Sección XVI, Tlalpan, 14000 Mexico City, DF, Mexico; ^3^Instituto Nacional de Cancerologia, Infectious Diseases Department, Avenida San Fernando No. 22, Col. Sección XVI, Tlalpan, 14080 Mexico City, DF, Mexico

## Abstract

*Background*. Universal access to antiretroviral therapy (ARVT) started in Mexico in 2001; no evaluation of the features of ARVT prescriptions over time has been conducted. The aim of the study is to document trends in the quality of ARVT-prescription before and after universal access. *Methods*. We describe ARVT prescriptions before and after 2001 in three health facilities from the following subsystems: the Mexican Social Security (IMSS), the Ministry of Health (SSA), and National Institutes of Health (INS). Combinations of drugs and reasons for change were classified according to current Mexican guidelines and state-of-the-art therapy. Comparisons were made using *χ* ^2^ tests. *Results*. Before 2001, 29% of patients starting ARVT received HAART; after 2001 it increased to 90%. The proportion of adequate prescriptions decreased within the two periods of study in all facilities (*P* value < 0.01). The INS and SSA were more likely to be prescribed adequately (*P* value < 0.01) compared to IMSS. The distribution of reasons for change was not significantly different during this time for all facilities (*P* value > 0.05). *Conclusions*. Universal ARVT access in Mexico was associated with changes in ARVT-prescription patterns over time. Health providers' performance improved, but not homogeneously. Training of personnel and guidelines updating is essential to improve prescription.

## 1. Introduction 

The use of highly active antiretroviral therapy (HAART) has shown a dramatic impact in terms of decreasing morbidity and mortality among HIV/AIDS patients [1]. However, the large-scale implementation of antiretroviral therapy (ARVT), a term used to describe any type of antiretroviral drug regimen, has faced enormous barriers worldwide, particularly in middle and lower income countries. These obstacles are not solely related to the high cost of antiretrovirals but also to other factors related to the health systems and access to drugs. Some of these barriers have been as follows: a low level of expertise of the prescribing health professionals, little investment in training of healthcare workers, and limited laboratory capacity for viral load (VL) and CD4 monitoring [2]. Other more complex factors, such as poor adherence to treatment, antiretroviral drug toxicities, emergence of viral resistance, and social factors that include incarceration, drug abuse, and unmet basic human needs caused by extreme poverty, have also been mentioned as barriers to optimal treatment effectiveness [3, 4].

In Mexico between 1997 and 2001, ARVT was only provided to subjects that were affiliated with the Instituto Mexicano del Seguro Social (IMSS), Mexican social security medical institutions, which only covers individuals who are employed in the formal sector and their dependents. Universal access to ARVT for the rest of the population (approximately 50%) was incorporated in 2001 by the Mexican government, although the policy was only fully implemented in 2003. Before this, access to ARVT for non-IMSS affiliated patients was limited and not sustained, which significantly impaired optimal adherence to ARVT and made it difficult if not impossible for physicians to comply with national treatment guidelines, and thus a large proportion of HIV patients undergoing treatment were exposed to suboptimal antiretroviral therapies [5].

Along with the large investment made by the government to improve access, an enormous effort was made to elaborate and update national guidelines for antiretroviral prescriptions, the first of which were published in 1997. However, at the individual level, prescriptions were not necessarily based on those guidelines but were influenced by other factors such as shortages, health provider expertise, and the impact of marketing pressure on prescribing physicians. While there is no evidence of these types of impacts in Mexico, they have been documented in other high- and middle-income countries [6, 7].

A previous study during the initial period of the universal access program in Mexico showed a widespread pattern of inadequate antiretroviral prescriptions in a convenience sample of facilities in the three subsystems [8]. To our knowledge, no additional assessment of the universal access program has been carried out [8, 9] nor has any other study assessed changes in the adequacy of antiretroviral prescriptions in Mexico over time [10]. The objective of this study was to assess the adequacy of antiretroviral prescriptions—in terms of physician adherence to national guidelines or the state of the art in HIV medicine—in four Mexican clinics illustrative of the country's three main health subsystems before and after implementation of the universal access program.

## 2. Methods 

### 2.1. Setting

The study that this paper is based on was a pilot study funded by the University of California Institute for Mexico and the United States (UC MEXUS) in 2005. The objective of this pilot study was to provide data to inform both decision-making and a future NIH grant as a proof of concept project. In particular, one specific objective of the project was to assess “the variability of compliance by care providers with national treatment guidelines.” The sample size determined for the original study protocol was 625, which represented 90% of the total number of patients eligible to receive HAART in the sampled facilities in 2002, proportionally distributed across facilities. The selection of 643 patients was random in order to ensure representativeness. Study subjects were selected from a list of “active” patients in each facility in 2002. We ordered this list in an Excel spreadsheet and then selected every other 4th, 5th, or *n*th depending on the sample size in each facility.

Information was collected retrospectively from the medical records of the 643 randomly selected HIV-infected patients at four healthcare facilities between 2001 and 2005. Patients from each center were stratified as follows: 75% of patients on ARVT, 10% deceased patients, and 15% not eligible for treatment. For the purpose of the study, only subjects who initiated antiretroviral therapy within the first 3 months after beginning care were included in the analysis. The facilities included in this study were selected for convenience to represent the spectrum of care provided in Mexico and included (1) a second-level hospital of the Mexican Social Security System (IMSS), where patients were treated by a single internist, and antiretrovirals were available for the entire period of the study; (2) a second-level hospital of the Ministry of Health System (SSA), Hospital General de Cuernavaca (HGC), which offers care for the uninsured population, and where patients were treated by a general practitioner, and (3) two third-level, academic hospitals that are part of the National Institutes of Health (Institutos Nacionales de Salud—INS), Instituto Nacional de Ciencias Médicas y Nutrición Salvador Zubirán (INCMNSZ), and Instituto Nacional de Cancerología (INCAN), which are specialized institutes catering to the general population and the two oldest AIDS clinics in Mexico, where patients were treated by infectious disease specialists.

Data extracted from the records included date of treatment initiation, history of drugs prescribed, CD4 counts, and VL tests performed over time. Our data covers retrospective information starting from 1990 for INCAN and INCMNSZ, 1993 for the IMSS, and 1999 for the SSA, the dates when HIV/AIDS clinics were formally initiated in each institution, and continuing through mid-2005 in all cases.

Antiretroviral prescriptions were assessed in terms of their adherence to national guidelines by two of the authors, (PV-YC), an infectious disease specialist with 20 years of experience in HIV care and the principal author, who were blinded to the patient affiliation. All antiretroviral combinations ever prescribed to our sample of patients were classified in one of the following categories:

- *adequate* because they were explicitly recommended in the national guidelines that were current in the year of use or indicated according to the current state of the art,
- *inadequate* because the combination of drugs was explicitly contraindicated, was a toxic one, increased the risk of adverse reactions, was antagonistic, and was not recommended due to demonstrated lower efficacy in clinical trials or because no clinical trial had documented the efficacy of the regimen.

All ARV treatment changes were classified according to the reason for change, which was evaluated by the authors according to information from patient records, into one of the following categories:

- *presumed viral failure*, assumed when the new regimen included two new drugs, one of which was of a different class, even though this was not always documented by a detectable VL (VL was not available in all facilities, and, for three facilities, patients had to pay for the test when it became available in 1997),
- *toxicity*, assumed when one drug from a group was substituted for another of the same group in a patient with suppressed viral load,
- *optimizing a previous suboptimal regimen*, that is, when mono- or bitherapy was changed to a 3-or-more drug regimen or when a less valid regimen was changed to a valid regimen according to prevailing recommendations at the time,
- *deemed inadequate by existing guidelines*, that is, when the new combination was more toxic or antagonistic or when no study had been published using this combination. The proportion of regimens in each facility falling into either adequate or inadequate antiretroviral combination categories and into categories describing the four different reasons for changing regimen was evaluated over the indicated periods of time. The first three categories of reasons for change were considered adequate and the last was inadequate.

### 2.2. Periods of Analysis

Antiretroviral prescription adherence to prevailing guidelines or norms was evaluated before and after 2001 and classified as described above. The official guidelines or norms selected as references had to have been published at least 3 months before the evaluated prescription.

*Before universal access*, until 2000, we used the official norm NOM 003-SSA2-1993 [11] and “the guide for care of patients with HIV/AIDS in outpatient services and hospitals,” published in May 1997 [12]. *After universal access*, since 2001, we used “the guide for care of patients with HIV/AIDS in outpatient services and hospitals, second edition” published in June 2000 [13]. Bitherapy was still accepted as adequate in these guidelines, which were valid until December 2003. “The guide for antiretroviral treatment for people who live with HIV/AIDS [14]” was published in December 2003. This was the first national guideline that specifically considered antiretroviral bitherapy inappropriate. During both periods, all prescriptions recommended in the current international guides were considered adequate.

### 2.3. Statistical Analysis

Differences between facilities in the type of therapy initiated during the studied time period were estimated using a *χ* ^2^ test. To compare the distribution of combination drugs prescribed in each facility by period, we used a *χ* ^2^ test. To estimate the probability of prescribing appropriately, we used a logit model controlling for period and facility. We compared the proportions of changes by reason between facilities using a *χ* ^2^ test. A type I error probability of 0.05 was used as the threshold for statistical significance. Statistical analyses were performed using STATA version 10.

## 3. Results 

### 3.1. Patient Characteristics

**Figure 1:**
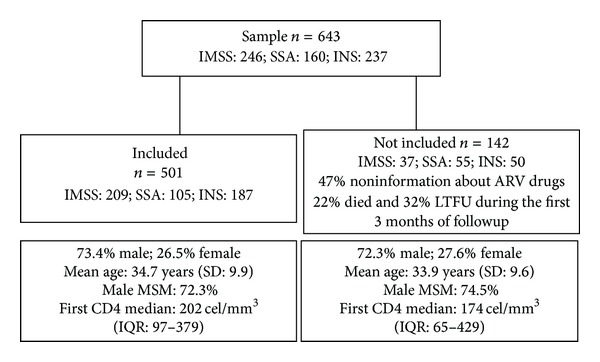
Comparison between patients who were included and patients who were not included.

The total number of patients in the sample was 643, 246 of which were from the IMSS, 160 from the SSA, and 237 from the INS. Of the 643 individuals in the original database, 142 (37 from the IMSS, 55 from the SSA, and 50 from the INS) were not included in the analysis for one of the following three reasons: 47% of their records did not have information on antiretroviral drugs, 22% died within three months of beginning care, and 32% were lost to followup (LTFU) within three months of beginning care. Patients not included and patients included in the analysis had similar distributions in age at initiation of care (*P* = 0.48), sexual preference (*P* = 0.36), first CD4 count (*P* = 0.78), and gender (*P* = 0.79), but there were significant differences in the facility they attended (85% of patients were included from the IMSS, 66% from the SSA, and 79% from the INS; *P* = 0.00) and the period of analysis (before 2000, 8% were not included and 92% were included, and, after 2001, 29% were not included and 71% were included; *P* = 0.00) (Figure 1).

The analytical sample was 501 patients, 78% of the original sample: 209 (41%) from the IMSS, 105 (21%) from the SSA, and 187 (37%) from the INS. The mean follow-up time from treatment initiation for the 501 included patients was 3.69 years (SD: 3.34) with a median of 2.69 years (IQR: 1.00–5.40). By subsystem, we found a mean of 3.47 years (SD: 2.69, range: 0.25–12.3) and a median of 3.0 years (IQR: 1.23–5.40) for the IMSS; a mean of 1.7 years (SD: 1.4, range: 0.25–5.6) and a median of 1.28 years (IQR: 0.59–2.62) for the SSA; and a mean of 5.06 (SD: 4.08, range: 0.3–15.4) and a median of 3.28 years (IQR: 1.49–9.05) for the INS. Follow-up times were significantly different between health subsystems (*P* value < 0.01).

### 3.2. Time of First Use of Drugs by Subsystem

**Figure 2:**
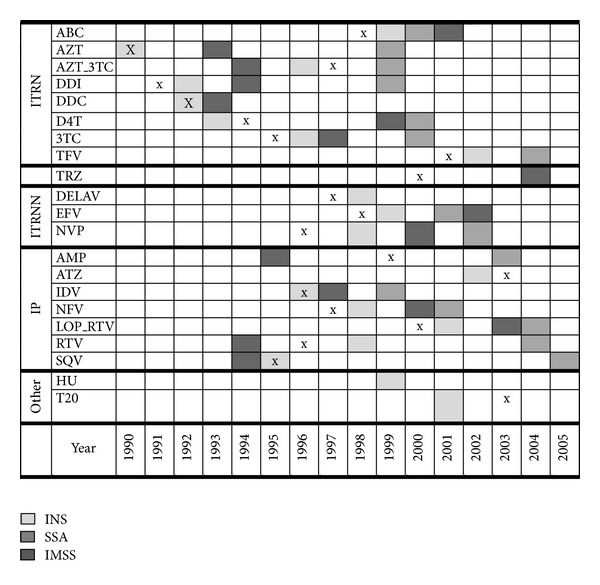
Type of drugs used by subsystem and year of introduction. *Note*. Every drug was used beginning at the year marked in the figure during the period of study, with the following exceptions in the INS: DELAV was used between 1998 and 1999, HU was used between 1999 and 2000, and DDC was used between 1992 and 1998. The “x” indicates the year the FDA approved the drug, except for AZT which was approved in 1987 [15].

The time of use of specific antiretroviral drugs in each subsystem could be an indicator of the adequacy of the drugs prescribed. In this study, early use of nucleoside analog reverse transcriptase inhibitors (ITRN) was observed in all facilities (Figure 2). The first drug used in monotherapy was AZT in 1990 in the INS. In the IMSS, the first ARVT used was DDC+AZT in 1993 and monotherapy with AZT in 1994. The SSA started with IDV+AZT+3TC in 1999. For nonnucleoside reverse transcriptase inhibitors (NNRTI), the IMSS began NVP use in 2000 and EFV in 2002. In the SSA, EFV was first prescribed in 2001 and NVP in 2002. The INS reported the use of DELAV and NVP beginning in 1998 and EFV in 1999. For protease inhibitors (PI), the IMSS reported its use beginning in 1994, the SSA in 1999, and the INS in 1995. In Mexico, PI use before 1997 was always through pharmaceutical-sponsored clinical trials. Other drugs used only for HIV therapy in INS facilities were hydroxyurea between 1999 and 2003 and enfuvirtide between 2001 and 2005 under research protocols. Figure 2 shows the first year each drug was used by every subsystem in Mexico and the year each drug was approved by the US Food and Drug Administration (FDA) [15]. The time between FDA approval and first use of the reported drug was shorter in each group of drugs in the INS compared to the IMSS and the SSA. In some cases, the year reported matched the FDA approval year or earlier, most likely because facilities participated in clinical trials, which could be interpreted as an intention to update and improve prescriptions.

### 3.3. Combinations of Drugs by Type and Period

**Figure 3:**
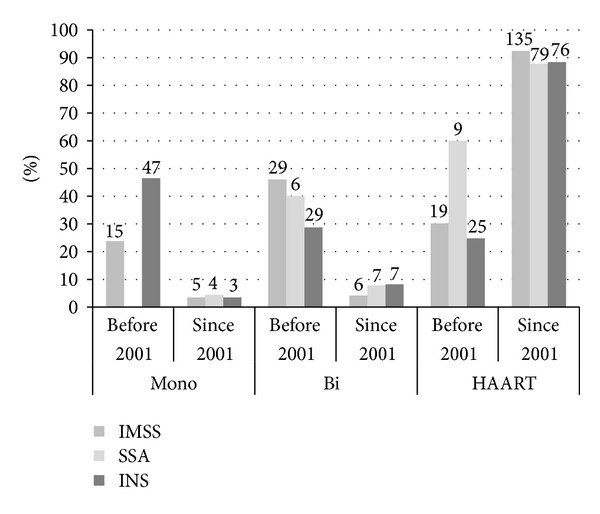
Distribution of patients by health subsystem and the type of initial ARVT. *Percentages were calculated with respect to the number of patients initiating therapy by period in each facility. IMSS information was available from 1993, SSA from 1999, and INS from 1990. The number over each bar is the total number of patients by category.

Numbers and percentages of patients by type of antiretroviral therapy (monotherapy, biotherapy, and HAART) facility and period are shown in Figure 3. Thirty-seven percent of the patients (*n* = 62) starting ARVT before 2001 were treated with monotherapy, compared to 3.6% (*n* = 12) after 2001. The drug used in most of the cases was AZT (70%), followed by DDI (16%). Twenty-nine percent of patients who started antiretroviral therapy before 2001 and 90% after 2001 used a regimen with 3 or more drugs. Before 2001, there were differences between the percentages of patients using HAART in each subsystem: IMSS versus INS (30% versus 25%, *P* value = 0.01), SSA versus INS (60% versus 25%, *P* value = 0.000) and IMSS versus SSA (30% versus 60%, *P* value = 0.03). After 2001, no significant differences were found between the SSA and the IMSS (87% versus 92%, *P* value = 0.41), nor between the IMSS and the INS (92% versus 88%, *P* value = 0.44) and the SSA and the INS (87% versus 88%, *P* value = 0.99).

The percentage of adequate antiretroviral regimen combinations that were started decreased between the two periods of study in each facility. In the IMSS, 75% of prescriptions were adequate until 2000 versus 38% after 2001 (*P* value < 0.001). The percentage also decreased from 100% to 72% (*P* value = 0.02) in the SSA and from 94% to 79% in the INS (*P* value = 0.02) (Table 1). Significant differences were found between facilities in each period (*P* values = 0.001 and 0.000, resp.).

In a logit model controlling for subsystem and period, a higher probability of an initial adequate prescription was observed in the INS and the SSA compared to the IMSS (*P* value < 0.01), and all facilities had a higher probability of an adequate prescription before universal access (*P* value < 0.01 logit model) (Table 2).

### 3.4. Changes in Treatment by Category and Period

Table 3 shows the distribution of changes by reason for change, facility, and period. Comparisons between facilities by reason for each period are included, as well as a general comparison between facilities including all reasons for each period. In general, most of the changes were evaluated as adequate; only 6.5% of the total changes were considered inadequate (Table 3). The distribution of reasons for change between facilities was significantly different before 2000 and after 2001 (*P* values = 0.05 and 0.001, resp.).

Optimizing a previous suboptimal regimen was the first cause for change in both periods and equaled 40% of the total changes until 2000 and 46% after 2001. Until 2000, 45% of the changes occurred for this reason in the IMSS, 16.7% in the SSA, and 39.1% in the INS (*P* value = 0.22). After 2001, the percentages were 44.5%, 54%, and 44.4%, respectively (*P* value = 0.09). Differences between subsystems were found in the category of changes for presumed viral failure after 2001 (*P* value = 0.01) but not in the period up to 2000 (*P* value = 0.67). Changes for toxicity started early in facilities with more experienced physicians; the INS reported that 21% of changes were for this reason up to 2000, which was higher than the percentages in the IMSS (12.8%) and the SSA (16.7%). Differences were significant between the IMSS and the INS (*P* value = 0.03). After 2001, the percentage of changes for toxicity between all facilities were more similar (*P* value = 0.12). A significant difference was found when inadequate changes were compared between IMSS and INS facilities in each period; it was significantly higher in the IMSS than the INS in both periods (8.3% in the IMSS versus 3.9% until 2000, *P* value = 0.04 and 9.4% in the IMSS versus 3.8% in the INS after 2001, *P* value < 0.01). After 2001, the differences in changes for unknown reasons that were categorized as inadequate between the three subsystems were significant (*P* value = 0.00).

The percentages of changes for each reason were not significantly different during the studied time in the IMSS (*P* value = 0.97) or in the SSA (*P* value = 0.13), but it was marginally significant in the INS (*P* value = 0.07), where the percentage of changes for toxicity decreased after 2001 (21% versus 14.8%) and changes for optimizing increased (39.1% versus 44.4%). By facility between periods, no differences for presumed viral failure were found (IMSS *P* value = 0.68, SSA *P* value = 0.17, and INS *P* value = 0.74). The percentages of inadequate changes in each facility (IMSS and INS) were similar between periods (8.3% until 2000 versus 9.4% after 2001 in the IMSS, *P* value = 0.67 and 3.9% until 2000 versus 3.8% after 2001 in the INS, *P* value = 0.65) (Table 3).

Boosting PI with ritonavir was not considered a change of regimen, but it was found in 88 patients (49%), 12 before 2001 and 76 (24%) in the second period. The medical facility with the highest percentage of boosting was the INS, with 92% before 2001 and 51% for the second period.

## 4. Discussion

In this study, we documented a heterogeneous pattern of adequacy in ARVT prescriptions in the health facilities included. Our results showed a significant increase in the use of HAART after 2001, with a higher percentage in the IMSS compared to the INS and the SSA during the first period but with a similar distribution between facilities after 2001. A higher probability of an adequate initial prescription was observed in the INS and the SSA compared to the IMSS in both periods, especially before 2001. The distribution of reasons for changes to prescriptions was not significantly different during the study period in the IMSS or the SSA, but it was marginally significant in the INS, where the percentage of changes for toxicity decreased after 2001 and changes to optimize ARVT increased. A significant difference was found when inadequate changes were compared between facilities; it was significantly higher in the IMSS than the INS in both periods, but percentages in each facility (IMSS and INS) were similar between periods.

After the first local guidelines for antiretroviral drug prescriptions were published in 1997 [12] and after achieving universal access in 2001, the adequacy of prescribing ARVT according to local guidelines was attained in a great percentage of the facilities studied. However, according to the prevailing practice at the time, a high percentage of patients were exposed to suboptimal therapy even in facilities where access to HAART started in 1997, as guidelines were not updated with advances in HIV medicine.

Access to antiretroviral therapy in Mexico has been a stepwise process depending on the health subsystem. Each subsystem has different antiretroviral drug availability conditions that have had an impact on the ability of physicians to prescribe them. For social security workers, it became possible to receive a triple HAART regimen in 1997, even though only 30% of IMSS patients began receiving HAART before 2001. However, for the other hospitals included in this study, universal access started as a progressive process after 2001, when HAART was included in the Seguro Popular [16, 17], and thus every HIV patient with no social security and less than 350 CD4 or with an AIDS defining event was given free access to antiretroviral therapy.

Although guidelines were published for antiretroviral use [12–14], they were not mandatory and no mechanism for monitoring prescriptions existed. The diversity of antiretroviral prescriptions is wide, as is the experience of the prescribing physician in each hospital [8]. Similar patterns of treatment changes and initial antiretroviral prescriptions were observed in the facilities with more experienced physicians, the INS and the IMSS (Table 3). The facility that utilized general practitioner care for HIV patients, the SSA, tended to adhere more to guidelines for initial antiretroviral prescriptions (Table 1), although inadequate changes were higher among the group of patients treated by general practitioners after universal access (Table 3). This is a matter of concern as huge economical resources are spent to increase antiretroviral access, but these resources are far from optimal because the physicians prescribing them are undertrained.

Prescriptions before 2001 (universal access) were not necessarily the best possible ones due mainly to limited access to antiretrovirals. Of concern is that, before 2001 in the IMSS, all antiretroviral drugs were available free of charge, but the prescription patterns were similar to other facilities that did not have free access to antiretroviral drugs or monitoring tests during this period, and over time the impact on prescriptions after 2001 was similar to other institutions. This is a difficult phenomenon to explain, as we would expect that with full antiretroviral availability the prescriptions would be written according to guidelines. However, a decrease in the percentage of adequate combinations is plausible as it may be related to an insufficient supply of ART compared to an increasing demand explained by the expansion of universal access. Physicians may have prescribed older and outdated combinations because they were available in stocks. New combinations may have taken time to be widely available in the country. Another potential explanation is the lack of training to introduce physicians to the new guidelines. ARV shortages could cause the delivery of incorrect prescriptions [18], but we acknowledge that further studies are needed to explain it better, particularly in the case of the IMSS.

Our results show that patients had been exposed to a great extent to suboptimal therapy in the studied facilities before 2001. On average, 62% received monotherapy or bitherapy with nucleoside analogues during this period, even when international guidelines did not recommended monotherapy or bitherapy beginning in 1998 [19]. Mexican guidelines removed the recommendation for bitherapy in 2003 [13, 14], which was clearly outdated considering the already existing evidence and international guidelines at the time. This prolonged use of suboptimal therapy could have had an impact on patient outcomes and on the emergence and transmission of resistant virus in the community.

Mexican guidelines were written in the year 2000 with concepts that were far behind the state-of-the-art knowledge of antiretroviral therapy, which resulted in larger use of suboptimal 2 nucleoside regimens with the consequent selection of drug resistance and possible transmission in the community. A recent study of HIV transmitted drug resistance (TDR) for the period of 2005–2009 in Mexico showed a 7.4% prevalence of TDR HIV, which was higher than that predicted by WHO TDR surveillance. The prevalence of nucleoside reverse transcriptase inhibitors (NRTIs) in this representative cohort was 4.2%, the highest of all antiretroviral drugs, and, of these, 70% had thymidine analogue mutations (TAMs), and up to 20% had 2 or more TAMs [20]. The prevalence of TAMs was higher in Mexico compared to the USA (52% versus 36%) for the same study period [20]. These findings may be relevant in light of the recently published 2012 National Guidelines, which contain recommendations on first and second line treatments that are again not in line with other international guidelines [21].

Because mechanisms to update guidelines to reflect fast-changing current evidence do not exist in Mexico, the guidelines usually become obsolete rapidly, as reported in this study, with significant public health consequences. In contrast, we can argue that scientific knowledge is rapidly socialized and that the speed of socialization has been increasing for several decades and particularly in the last decade. However, physicians face different situations in their practice. They may have knowledge from different sources regarding what will be the best regimen for a patient to be prescribed. Also if the patient must pay for antiretrovirals out of their own pocket and does not have the economic resources to pay for triple therapy, then the physician had to assess this particular situation and make a decision. This was the situation for the INS and the SSA until 2001 when universal access was announced. However, again, this was a gradual process; every patient had to fulfill certain administrative requirements to be a candidate and beneficiary for universal ARV access.

This study shows that prescriptions that are written by physicians without experience in HIV medicine and who follow the guidelines strictly or are compelled to do so by authorities may have negative consequences. In the current environment, training medical doctors in nonspecialized facilities and improving access to laboratory resources (VL and genotype tests) are important for improving ARVT prescriptions. We envision two ways to improve prescriptions in our health system: (1) monitoring and supervision by peers of ARVT prescribed in facilities where physicians are less trained or less experienced and (2) restricting options for first line regimes for nonspecialized physicians.

The main limitation of our study is that it evaluates ARVT prescription patterns in only four facilities in the country, and although we cannot claim that the study sample represents the whole country, these four facilities are referral centers (special departments within a hospital or institution specializing in the care of HIV/AIDS patients) for HIV-patient care in each of the health subsystems. In addition although the facilities were selected for convenience, all the subsystems were represented. As explained before, the data were collected in 2005, which explains why we do not have information for subsequent years. The relevance of these results stems from the fact that we described the prescribing patterns before and after the introduction of a universal access, policy in a middle-income country with a fragmented health system, a wide heterogeneity in the prescribing physicians' clinical capacities and with fewer alternatives for prescriptions than there are currently. In the context of a significant global push for universal access to health care in general and to antiretrovirals in particular, significant lessons can be drawn from the experience in Mexico in the early 2000s.

Our work has other limitations common to retrospective studies; in addition to having a nonrepresentative sample of all clinics treating HIV patients in the country, we have no data regarding the supply of antiretroviral drugs during this time for each facility nor do we have data concerning the patients' adherence to antiretroviral regimens. All this would allow us to extrapolate our results to other Mexican facilities and to explain how patterns of prescriptions are affected by other circumstances besides physician expertise. Although this study does not measure patient outcomes, we were able to evaluate the adequacy of prescriptions according to the guidelines in place. Universal access did improve the appropriateness of prescriptions in facilities where patients paid out-of-pocket for their antiretrovirals before universal access, but it had no impact in the one facility where there had been full access since 1997 (IMSS).

The scaling-up of ARVT poses enormous challenges, aside from the economic burden of providing drugs and the high cost of laboratory exams; the success of this huge effort of governments and agencies may be hindered if the prescriptions for these regimes are inadequate. National guidelines should be updated according to the pace of knowledge and should always leave open the possibility of tailoring HIV prescriptions for special cases through peer review. The clinical care of people with advanced HIV disease, with opportunistic infections, malignancies, and/or viral failure, could be very complex and thus requires the knowledge of experienced physicians to reach better clinical outcomes, to manage drug interactions, to promote adherence, and to avoid resistance transmission [22].

## 5. Conclusions

This study evaluates ARVT prescription patterns over time in four facilities of three different Mexican health subsystems. The results contribute to the identification of trends and the quality of antiretroviral prescriptions before and after the first years of universal access in Mexico, discuss possible explanations for the observed prescription patterns, and propose ways to improve prescriptions. The expected benefits to patient survival and quality of life with novel therapies require, in addition to sustained drug access, good prescription practices by physicians. Medical doctors with a high level of training and experience are as important as access to drugs and laboratory exams to monitor therapy responses [23–28].

## Figures and Tables

**Table 1:** Percentage of adequate initial antiretroviral prescriptions according to prevailing guidelines.

	*N*	Until 2000	*N*	After 2001	*P* value*
		% (*n*)		% (*n*)	
IMSS	63	75 (47)	146	38 (56)	0.00
SSA	15	100 (15)	90	72 (65)	0.02
INS	101	94 (95)	86	79 (68)	0.02

*P* value**		0.001		0.00	

*P value of differences between periods in each subsystem.

**P value of differences between periods for all facilities.

**Table 2:** Logit model for adequate prescriptions by subsystem and periods of analysis.

Adequate	OR	SE	*z*	*P* > |*z*|	95% CI
SSA	6.121	1.848	6.00	0.000	3.38–11.06
INS	6.714	1.922	6.65	0.000	3.83–11.76
After 2001	0.160	0.048	−6.11	0.000	0.08–0.28

Note: the IMSS was selected as the reference category to compare the probability of an adequate prescription in the SSA and the INS.

**Table 3:** Reasons for therapy change by subsystem and period of analysis.

%	Until 2000				After 2001			
	IMSS	SSA	INS	*P* value^a^	IMSS	SSA	INS	*P* value
Total changes	133	6	404		476	148	547	
Optimizing a previous suboptimal regimen	45.1	16.7	39.1	0.22	44.5	54.0	44.4	0.09
Presumed viral failure	33.8	50	35.9	0.67	31.9	24.3	36.9	0.01
Toxicity	12.8	16.7	21	0.03	14.1	9.4	14.8	0.11
Deemed inadequate	8.3	16.7	3.9	0.04	9.4	12.2	3.8	0.00

*P*-value^b^	0.04				0.00			

^a^Until 2000, the SSA was not included in comparisons for each reason due to the sample size.

^b^The distribution of reasons for change between facilities in each period is significantly different.
